# An automated cell line authentication method for AstraZeneca global cell bank using deep neural networks on brightfield images

**DOI:** 10.1038/s41598-022-12099-3

**Published:** 2022-05-12

**Authors:** Lei Tong, Adam Corrigan, Navin Rathna Kumar, Kerry Hallbrook, Jonathan Orme, Yinhai Wang, Huiyu Zhou

**Affiliations:** 1 School of Computing and Mathematical Sciences, University of Leicester, Leicester, UK; 2 Data Sciences and Quantitative Biology, Discovery Sciences, AstraZeneca R&D, Cambridge, UK; 3 UK Cell Culture and Banking, Discovery Sciences, AstraZeneca R&D, Alderley Park, UK; 4 UK Cell Culture and Banking, Discovery Sciences, AstraZeneca R&D, Cambridge, UK

**Keywords:** Cellular imaging, Bioinformatics, Time-lapse imaging

## Abstract

Cell line authentication is important in the biomedical field to ensure that researchers are not working with misidentified cells. Short tandem repeat is the gold standard method, but has its own limitations, including being expensive and time-consuming. Deep neural networks achieve great success in the analysis of cellular images in a cost-effective way. However, because of the lack of centralized available datasets, whether or not cell line authentication can be replaced or supported by cell image classification is still a question. Moreover, the relationship between the incubation times and cellular images has not been explored in previous studies. In this study, we automated the process of the cell line authentication by using deep learning analysis of brightfield cell line images. We proposed a novel multi-task framework to identify cell lines from cell images and predict the duration of how long cell lines have been incubated simultaneously. Using thirty cell lines’ data from the AstraZeneca Cell Bank, we demonstrated that our proposed method can accurately identify cell lines from brightfield images with a 99.8% accuracy and predicts the incubation durations for cell images with the coefficient of determination score of 0.927. Considering that new cell lines are continually added to the AstraZeneca Cell Bank, we integrated the transfer learning technique with the proposed system to deal with data from new cell lines not included in the pre-trained model. Our method achieved excellent performance with a precision of 97.7% and recall of 95.8% in the detection of 14 new cell lines. These results demonstrated that our proposed framework can effectively identify cell lines using brightfield images.

## Introduction

Over the last 50–60 years, cell lines have become a staple of biological research, resulting in rapid developments in the fields of cell and molecular biology, however, this increased use of cell lines has brought to light the issue of cell line authentication^1^. Many isolated cell lines were subsequently found to be contaminated by faster growing cell cultures, such as HeLa cells, due to poor cell culture practice leading to the misidentification of cell lines^1^. The use of a misidentified cell line can lead to false conclusions and irreproducible experiments, consequently leading to a waste of time, money and resource^2,3^. It is estimated that industry wide 10–20% of preclinical effort was wasted due to misidentified cell lines, estimated to cost the industry 28 billion USD per year^4^.

Though there are many methods for cell line authentication, short tandem repeat (STR) profiling, also sometimes referred to as DNA fingerprinting, has been the most widely used and is recommended as the standard by the American Type Culture Collection (ATCC) Standards Development Organization Workgroup ASN-0002^5^. In spite of the prevalence of STR profiling, there are limitations. Microsatellite instability and loss of heterozygosity, especially in cancer cell lines, can make validation and authentication challenging using STR profiling^6^. In one study involving hematopoietic cancer cell lines it was found that the effect of long term culture, subcloning, and selection led to genetic drift, thereby significantly altering the DNA fingerprinting and over time some cell lines may also go through genetic and transcriptional evolution (GTE)^7,8^. All of these variances can make it harder to discriminate between cell lines using STR profiling^8^. In addition, due to time and cost restrictions, standard practice is to test the cells once they have been fully expanded and frozen, however this process results in wasted time and effort if the sample then fails STR profiling. It was therefore desirable to develop a new authentication approach to complement STR testing. Ideally, the new method should be easy to use, quick, and cost effective, and would enable early identification of the cell line that could be built into standard laboratory practice as well as overcoming the limitations of STR profiling^9,10^.

With the advancement of machine learning (ML) approaches, automated cell image classification is a possible solution to allow fast analysis at low cost, as well as potentially identifying changes in cell morphology that could be indicative of undesirable qualities such as genetic drift or cellular senescence. Ponomarev et al. extracted 17 dimensional features (e.g. hole number in cell nuclei, average cell size) from stained ANA Hep-2 cell images^11^. They used a SVM classifier to learn the extracted feature vectors and got 70.57% accuracy on the 6-class classification of ANA Hep-2 cells. Murphy et al. utilized Zernike moment, object finding and edge detection to describe subcelluar location patterns of Hela cells which are labeled with antibodies against 10 endoplasmic reticulum protein separately^12^. Their proposed method achieved 83% accuracy on the classification task. Abdullah et al. collected 350 white blood cell (WBC) images and applied cell segmentation to extract geometric properties such as cell shape, nucleus size^13^. They compared 6 conventional ML classifiers on the classification of WBC images and reported Multinomial Logistic Regression algorithm outperformed other methods. However, the conventional machine learning methods rely on the pre-determined feature engineering like extracting morphology features from segmented cells. And such feature engineering methods should be adjusted according to the specific data^14^.

Deep learning approaches brings up the possibility of extracting the discriminative features from data without the need of the pre-determined feature engineering. Oei et al. collected 522 fluorescence cell images from 3 cell lines (e.g. MCF10A, MCF-7, MDA-MB-231) using confocal immunofluorescence microscopy^15^. By using the features of actin cytoskeleton structures, they proposed a convolutional neural network (CNN) based on VGG-16 to classify the microscopy images into three categories and reported the performance of CNN outperforms their biological experts in the classification task. Akogo et al. used MobileNet to perform image classification in their 5 cell line dataset (MDA-MB-468, MCF7, 10A, 12A and HC11) with 96.67% accuracy^16^. Most recently, Mzurikwao et al. trained two CNNs to classify 4 cancer cell lines (COLO 704, UKF-NB-3, EFO-27 and EFO-21) and their isogenic cell lines using brightfield images^10^. Compared with STR profiling, deep learning methods improve the cost and efficiency as these methods only need cellular images to train CNNs and the trained models are used to directly predict the identities for held-out test data. However, because of the lack of centralized available datasets and the intrinsic difficulties in analyzing multi-batch cellular images from different cell lines over time, the previous studies only conducted their experiments on small-scale cell line datasets of no more than ten cell lines. Whether or not CNN can identify tens even hundreds of cell lines is an open question. The relationship between the incubation times and cellular images has not been analyzed in previous studies. In addition, new cell lines are continually added to the AstraZeneca Cell Bank therefore, how to deal with data from new cell lines not included in the model requires consideration.

Therefore, in this study, we aim to answer these questions by developing an automated cell line authentication method by using deep learning-based analysis of routine brightfield cell line images. Our main contributions reported in this paper are:

1. Dataset: We have established two cellular datasets. i) The first dataset consists of 47,671 brightfield images of 30 cell lines, which is 23 GB of data. The dataset was curated from a set of experiments where 30 cell lines were cultured and each cell culture flask was imaged at 2–3 h intervals using the IncuCyte. To our knowledge, it is one of the largest such collections of data in the literature for cell line authentication. ii) The second dataset includes 860 cell images from 14 new cell lines not included in the 30 above. The 14 cell lines were incubated by using the same conditions as the previous 30 cell lines and we used this dataset to validate whether or not our proposed method can identify new cell lines without training model from scratch.
2. Cell image recognition framework: We proposed a novel multi-task cell image recognition framework to (i) identify and authenticate cell lines and (ii) predict the duration of how long cell lines have been incubated simultaneously. The cell line classification network (CLCNet) learns image-level features from the cell images and outputs the predicted probabilities of the cell line labels for each input image. The extracted convolutional features of CLCNet are integrated with the cell line regression network (CLRNet) to predict the incubated time points for bright-field images.
3. Identify new cell lines: We integrated a transfer learning approach with the proposed framework to identify images from new cell lines which are not included in the pre-trained model (currently we have 30 cell lines). This is an important aspect, as we are unlikely to be able to acquire very large numbers of images for every cell line that we wish to authenticate.
4. Validation: We conducted comprehensive validation experiments to justify the significance of our proposed framework on the established datasets. The prediction performance of CLCNet reached the accuracy of 99.8% and the f1-score of 99.7% in the classification of the 30 cell lines. CLRNet achieved the coefficient of determination score (R2-score) of 0.927 in the prediction of the incubated times for cell images. In the detection of 14 new cell lines, our proposed method obtained the precision of 97.7% and the recall of 95.8%. These results demonstrated that our proposed framework can effectively authenticate the identities of cell lines from brightfield images.

## Materials and methods

### Cell culture and image acquisition

**Figure 1:**
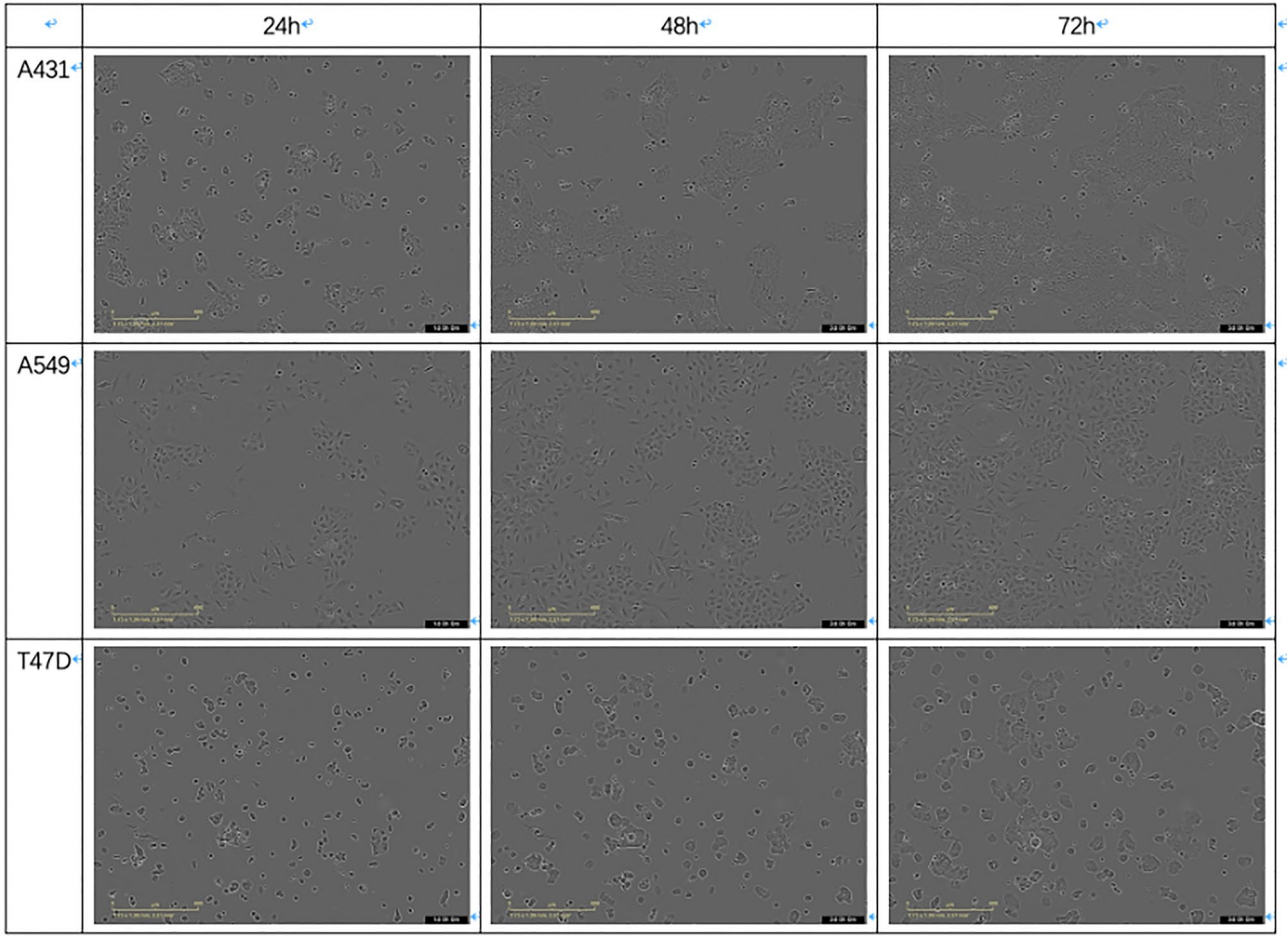
Example images of three cell lines with different incubated durations (hours). In the three examples shown here, 3 discrete time points were taken for A549, A549, T47D at 24, 48 and 72 h. With the increased amount of the incubation time, we observed increased cell counts and confluency and a formation of colonies. Notable single cell morphology can also be observed, e.g. A549 cells are more elongated in shape compared to A431 cells, whereas T47D cells are typically larger in size and round.

In this study, we have collected sample images from the registered cell lines in AstraZeneca Cell Bank where the selection criteria for cell types is “commonly used cell lines” based on the frequency of cell lines has been requested and the readily availability of corresponding images. We have established two cell line datasets. (1) The first dataset consists of 30 different cell lines, as listed in Table S1. The base medium is listed in the table, all medium was supplemented with 10% Foetal Bovine Serum (Sigma) and 1 × GlutaMAX (Gibco) unless otherwise stated. The cells were thawed and seeded into a 25cm^2^ flask (Corning) at a density of 0.5–2 × 10^6^ cells per flask. The flasks were then added to the Incucyte S3 (Essen Bioscience, Sartorius) system and brightfield images collected from 21 different locations across the flask every 2–3 h over a time period between 3 and 7 days. (2) The second dataset includes images from 14 cell lines, as listed in Table S2, these images were not used in the training of the CNN network. In this dataset, each cell line includes one location only resulting in fewer images. All images were exported for analysis as JPEG or TIFF format using the Incucyte S3 software with 1408 $\times$ 1040 size (96 $\times$ 96dpi). Example images of three cell lines (e.g. A431, A549, T47D) with different incubated durations are shown in Fig. 1.

### Multi-task framework overview

**Figure 2:**
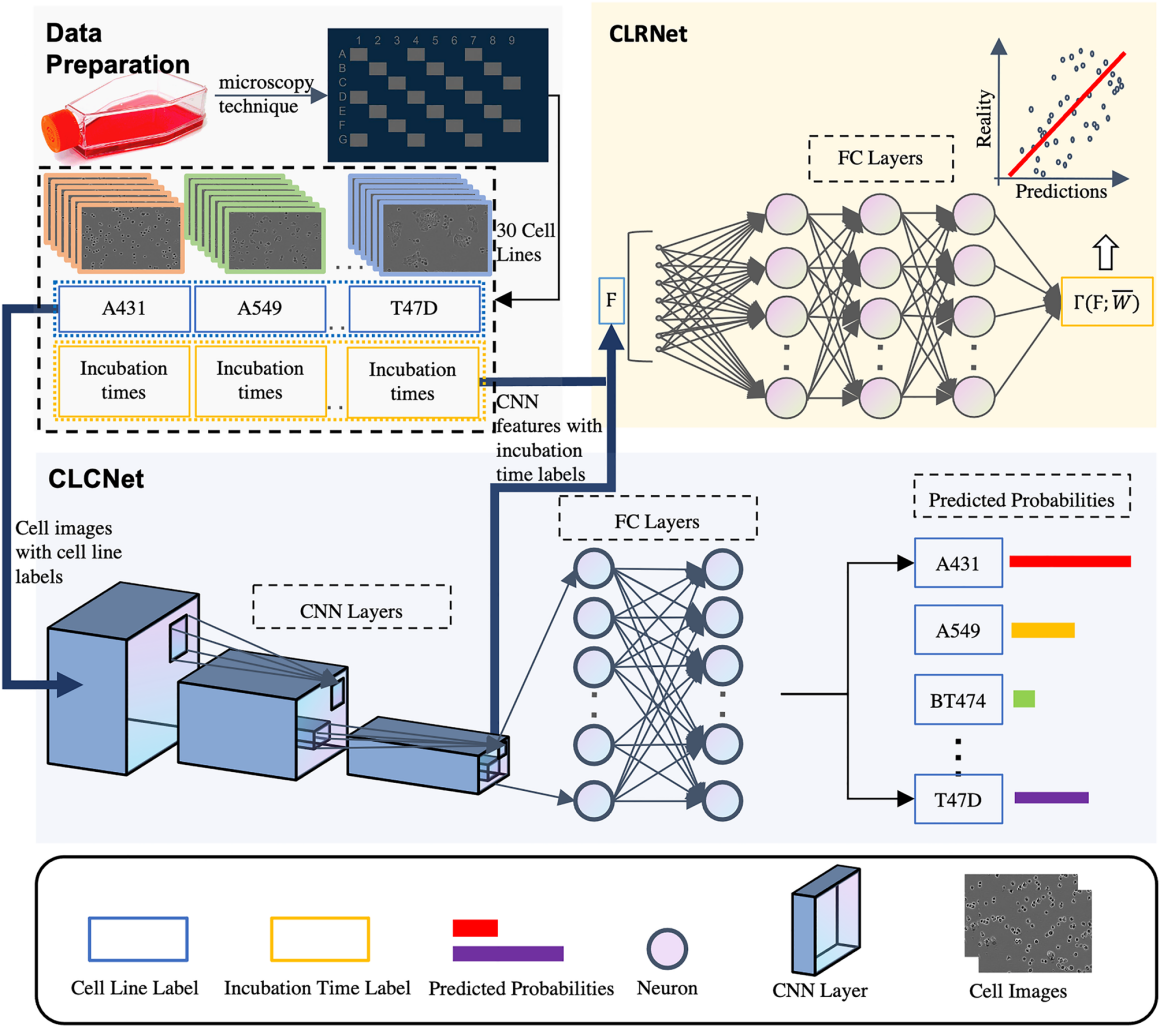
The framework of the proposed automated cell line authentication system. In the data preparation stage, cell images are collected using the high-throughput IncuCyte microscopy technique from 30 cell lines and each cell image has two separate labels, e.g. cell line name and incubation time. Then, the deep learning network CLCNet learns the image-level features from the input cell images with their cell line labels and outputs predicted classes for test cell images. Once CLCNet is trained, the convolutional features of the training data are extracted to train CLRNet. CLRNet predicts the times of how long cell lines have been incubated simultaneously.

Our proposed framework is shown in Fig. 2. In the data preparation stage, cell images are collected using the IncuCyte for all 30 cell lines. Each cell image has two separate labels, cell line name and incubation time. Then, the deep learning network CLCNet learns the image-level features from the input cell images with their cell line labels and outputs the predicted classes for test cell images. Once CLCNet is trained, the convolutional features of the training data are extracted to train CLRNet. CLRNet predicts the time of incubation after initial seeding for each image.

### Data pre-processing

Before the model training of CLCNet, we carried out the following pre-processing procedures: (1) Unify image format: The collected images’ formats include JPEG and TIFF. To ensure the consistency, we have unified all images’ formats to JPG before further processing for this study. (2) Image scaling: The original image resolution for the established dataset is 1408 $\times$ 1040. In the training dataset, each cell image is cropped at a random region with the size 896 $\times$ 869 to improve the data diversity, which can influence the generalization and robustness of the training model in the downstream tasks. Images from the test set are cropped at a fixed center region with the same size 896 $\times$ 869 for later method comparison experiments. The cropped images are resized to 224 $\times$ 224 using the bilinear interpolation^17^. (3) Grayscale: The scaled cell images are gray but have three channels. The grayscale method is applied to convert the images into one channel. (4) Data Normalization: Data normalization aims to restrict the image pixels within a specific range and ensure each pixel has similar data distribution^10^. Each cell image is normalized by subtracting the image mean (i.e. pixel values) and dividing by image standard deviation, to improve the convergence speed while training the neural networks.

### Cell line classification network

We employed the Xception model^18^ as the backbone of CLCNet for classification. The model structure used in our system is shown in Fig. S1. After pre-processing procedures, the input shapes of the cell images are 224 $\times$ 224 $\times$ 1 (Height $\times$ Width $\times$ Channel). The input images will first pass through two convolution layers that each convolutional layer is followed by batch normalization^19^ and a ReLU activation function. Five depthwise separable convolutional (DSC) blocks are stacked to further learn spatial and hierarchical features from the inputs. Each DSC block includes two or three DSC layers and one maxpooling layer. DSC layer consists of a depthwise convolution (i.e. a spatial convolution performed independently over every channel of input) and a pointwise convolution (i.e. a convolution with 1 $\times$ 1 kernel, projecting the channels computed by the depthwise convolution onto a new channel space)^20^. The mathematical formulation is defined as:

1 $$\begin{array}{c}{O}_{\text{k,l,E}} \, \text{=} \, \sum_{m}^{M}{\tilde{K }}_{m,E} \cdot \, \sum_{i,j}^{I,J}{K}_{i,j,m} \odot{F}_{i+k,j+l,m}\end{array}$$

where $\text{O}$ is the output feature map, $\text{k }\times \, l$ is the kernel size of the depthwise convolution and E is the channel of the output feature map. On the right -hand side of Eq. (1), $\tilde{K }$ and $K$ are the convolutional filters of the pointwise and depthwise convolution separately. $F$ is the input feature map and I $\times$ J $\times$ M is the shape of the input feature map. $\odot$ represents the operation of an element-wise product. The output of the DSC blocks is concatenated with the output of a shortcut convolution (1 $\times$ 1 Conv) through the residual connection. Only the fourth DSC block’s feature map concatenates with the input feature map without the convolutional processing. The dimension of the output feature map from the final two DSC layers is 7 $\times$ 7 $\times$ 2048 and the feature map is converted to 2048 dimensions by the adaptive average pooling layer. The final fully connected layer outputs the probability distribution for 30 cell lines. The classification loss ${\mathcal{L}}_{clc}$ is computed using the cross entropy:

2 $$\begin{array}{c}{\mathcal{L}}_{clc}= -\frac{1}{C}\sum_{c=1}^{C}\frac{1}{B}\sum_{{X}_{b}\in X}1\left\{{\Upsilon }_{b}^{c} =c\right\}\mathrm{log}(\mathrm{\rm P}({\Upsilon }_{b}^{c}=c|{X}_{b};W)) \, \end{array}$$

where $C$ is the number of class and $B$ is the batch size. ${X}_{b}$ represents the $b$-th sample in the batch and $1 \{\bullet \}$ denotes a characteristic function that $1 \left\{\bullet \right\}=1$ if the condition is true and 0 otherwise. $\mathrm{\rm P}({\Upsilon }_{b}^{c}=c|{X}_{b};W)$ is the probability of the sample ${X}_{b}$ being correctly predicted as the class ${\Upsilon }_{b}^{c}$ using the network parameters $W$.

During the network training, 20% of data are split from the training set for validation. The performance of CLCNet on the validation set is monitored for each five training epochs. Beyond epoch 50, if the validation loss has not decreased for ten consecutive epochs, early stopping is triggered and the best model with the lowest validation loss is used for reporting the performance on the held-out test set.

### Cell line regression network

Network features $F$ are extracted from the adaptive average pooling layer and its dimension is 2048. We employed a multilayer perceptron (MLP)^21^ as the backbone of CLRNet to refine features and reduce the dimension finally predict the incubation times for cell images. The network consists of three FC layers. The first two layers include 512 and 128 neurons separately and each layer is followed by a ReLU activation function. The third FC layer has 16 neurons and its output feature vectors are then transformed to a scalar which is the prediction result of the regression task. We add dropout units^22^ with 0.5 rates after all FC layers to avoid overfitting. The regression loss ${\mathcal{L}}_{clr}$ is computed using the mean squared error (MSE):

3 $$\begin{array}{c}{\mathcal{L}}_{clr}= \frac{1}{B}\sum_{{\mathrm{F}}_{b}\in F} ({\mathrm{\rm T}}_{b}-\Gamma ({\mathrm{F}}_{b};\overline{W}){)}^{2} \, \end{array}$$

where ${\rm T}_{b}$ is the ground truth and $\Gamma ({F}_{b};\overline{W})$ is the predicted incubation times for cell images. The incubation times of all cell images are converted to just hours.

The training of CLRNet starts when the training of CLCNet is done. We freeze the weight updating of the whole CLCNet. CLCNet acts as a feature extractor, and it outputs the feature vectors from the adaptive average pooling layer for the training and validation sets. The feature vectors of the training set are used to train CLRNet. The validation MSE loss is monitored each epoch and the best model with the lowest validation loss is saved after 50 epochs. It should be noted that CLRNet and CLCNet use the same training and validation data.

### Transfer learning for identifying new cell lines

To deal with data from new cell lines which are not included in the pre-trained model, combining the previous data with the new obtained data and retraining CLCNet from scratch is a way to solve this issue. However, the time and computational resources required are prohibitive for this approach. Hence, we integrated the transfer learning technique with CLCNet to identify new cell lines. Similar to the training strategy of CLRNet, we first take the pre-trained CLCNet model which is trained on the 30 cell lines dataset and freeze all layers except the last FC layer. The FC layer is replaced by a new FC layer with 44 neurons (i.e. 30 original cell lines + 14 new cell lines). The weights of the new FC layer are initialized. During the model training, only the weights of the FC layer will be updated with new data and the weights of other layers are fixed. The training set of the 30 cell lines is combined with the training set of the 14 cell lines. The underlying concept is that having been trained on 30 cell lines, the model will have learned a good representation of brightfield images for discriminating between different cell lines, and as such good classification performance can be obtained for new cell lines by only retraining the final classification layer. The combined test set is used to validate whether or not the updated model can keep good classification performance on the 30 cell lines and identify the new 14 cell lines.

### Ethical approval and consent to participate

The authors declare this study don does not require ethical approval.

## Results

### Implementation details

We implemented all models and benchmark experiments using Python 3.7 with Pytorch 1.9.0^23^ and Scikit-learn 0.24.0^24^ packages. The hyperparameters for CLCNet and CLRNet were set as follows: The SGD optimizer with the momentum = 0.9 and weight_decay = 5e−4 is adopted. The initial learning rate is 0.001 and it decays by factor = 0.1 every 25 epochs. The batch size and maximum training epochs are 20 and 100 separately. The two cell datasets were split into fivefolds separately then fivefold cross-validation (CV)^25^ was applied to evaluate the model performance. The CV iterated five times that fourfolds are used for training and validation, and the remaining fold for testing in each iteration. We deployed all the experiments using a single 32 GB Nvidia V100 GPU.

### Cell line classification and regression

**Figure 3:**
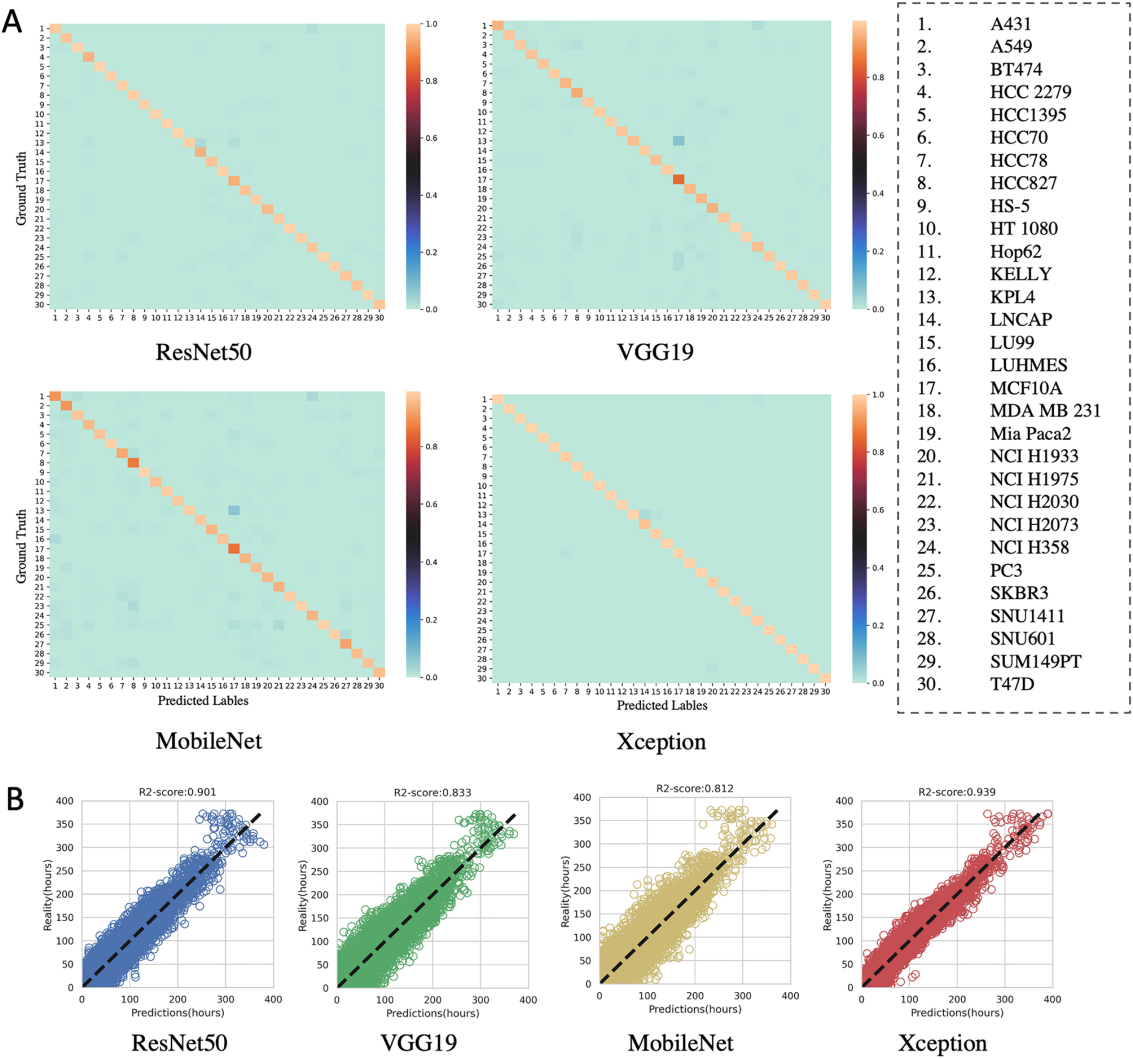
Visualization of the classification and regression results on the 30 cell lines’ dataset. (**A)** Confusion matrices of the 30-category classification. The corresponding cell line names of the coordinates are shown in the right legend. Compared with ResNet50, VGG19 and MobileNet, Xception model made fewer prediction errors. (**B)** Scatter plots of the predicted incubated durations vs. the real incubated durations. Here we show the prediction results on the test fold-1 as an example; the complete results of cross-validation are shown in Fig. S2. Xception outperformed other three methods in predicting the amount of incubation time with R2-score = 0.939.

In order to evaluate our proposed system comprehensively, we replaced the backbone of CLCNet with three SOTA CNNs: MobileNet, VGG19, ResNet50. To seek a fair comparison, the data selected for training, validation and testing are constant during the classification and regression tasks. The results of the classification and regression on the 30 cell lines are shown in Table 1. We evaluated the classification performance of CLCNet with four metrics, namely accuracy, precision, recall and f1-score:

4 $$\begin{array}{c}accuracy = \frac{TP+TN}{TP+FN+TN+FP}\end{array}$$

5 $$\begin{array}{c}precision = \frac{TP}{TP+FP}\end{array}$$

6 $$\begin{array}{c}recall = \frac{TP}{TP+FN}\end{array}$$

7 $$\begin{array}{c}f1= \frac{2\times \mathrm{precision}\times \mathrm{recall}}{\mathrm{precision}+\mathrm{recall}}\end{array}$$

where TP is true positive, TN is true negative, FN is false negative and FP is false positive. The Xception model obtained the best classification performance and achieved the average accuracy of 99.8%and the f1-score of 99.7% across the fivefold cross-validation. The classification features of the four backbones were extracted to train the CLRNet separately. The regression performance is evaluated by two metrics, MSE and R2-score:

8 $$\begin{array}{c}MSE= \frac{1}{\mathrm{N}} \sum_{\mathrm{n}=1}^{\mathrm{N}} ({\mathrm{Y}}_{\mathrm{n}}- {\widehat{\mathrm{Y}}}_{\mathrm{n}}{)}^{2}\end{array}$$

9 $$\begin{array}{c}R2 =1- \frac{\sum_{\mathrm{n}=1}^{\mathrm{N}} ({\mathrm{Y}}_{\mathrm{n}}- {\widehat{\mathrm{Y}}}_{\mathrm{n}}{)}^{2}}{\sum_{\mathrm{n}=1}^{\mathrm{N}} ({\mathrm{Y}}_{\mathrm{n}}- \overline{\mathrm{Y} }{)}^{2}}\end{array}$$

where N is the number of test samples. ${\mathrm{Y}}_{\mathrm{n}}$ is the real value of the nth sample and ${\widehat{\mathrm{Y}}}_{\mathrm{n}}$ is the predicted value. $\overline{\mathrm{Y} }$ denotes the average of all samples. The overall MSE and R2-score of Xception for the incubation duration prediction were 262.283 and 0.931 separately. We further demonstrated the classification results by using the confusion matrix in Fig. 3A, which shows that Xception made fewer prediction errors than ResNet50, VGG19, and MobileNet. The regression results of the test fold-1 are visualized by using the scatter plots, Fig. 3B. Xception outperformed the other three methods in predicting the amount of the incubation times with R2-score = 0.939. The complete regression results of the fivefold cross-validation are shown in Fig. S2.

**Table 1:** Classification and regression results on the 30 cell lines dataset: [mean value $\pm$ standard deviation] by four deep networks.

Backbones	Classification				Regression	
	Accuracy	Precision	Recall	F1-score	MSE	R2-score
ResNet50	0.987 $\pm$ 0.002	0.984 $\pm$ 0.002	0.987 $\pm$ 0.003	0.985 $\pm$ 0.002	452.533 $\pm 54$.547	0.880 $\pm 0$.015
VGG19	0.975 $\pm$ 0.004	0.968 $\pm$ 0.004	0.972 $\pm$ 0.003	0.972 $\pm$ 0.003	677.637 $\pm 43$.709	0.821 $\pm$.0013
MobileNet	0.965 $\pm$ 0.001	0.952 $\pm$ 0.002	0.960 $\pm$ 0.002	0.956 $\pm$ 0.002	769.111 $\pm 44$.984	0.797 $\pm$ 0.013
Xception	**0.998** $\pm$ **0.001**	**0.997** $\pm$ **0.001**	**0.997** $\pm$ **0.001**	**0.997** $\pm$ **0.001**	**262.283** $\pm$ **32.085**	**0.931** $\pm$ **0.008**

R2-score: coefficient of determination regression score. The best results are shown in bold.

**Figure 4:**
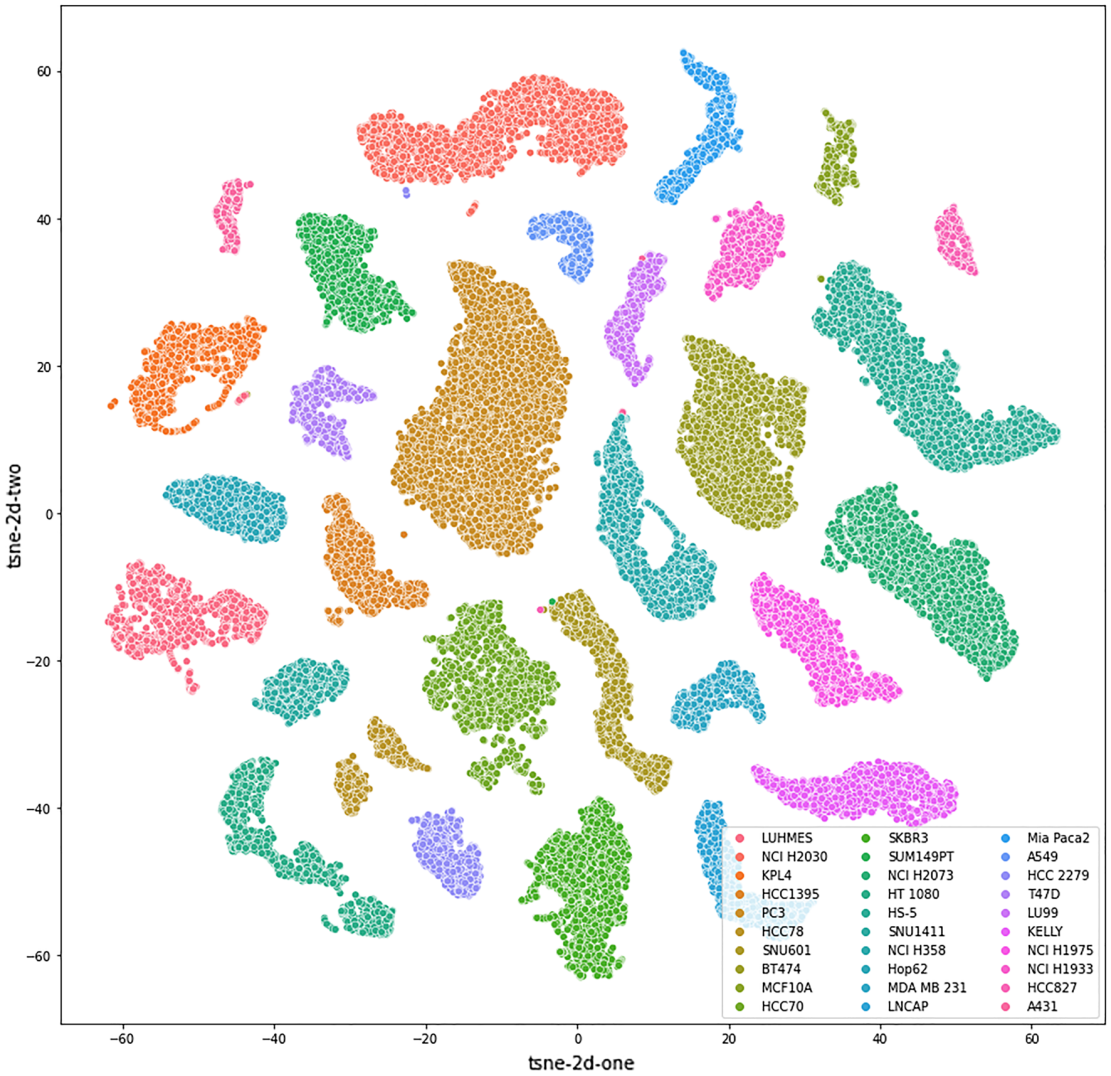
t-SNE Embedding of our 30 cell lines. The t-SNE tool reduces the dimension of the CLCNet’s convolutional features and visualizes the processed features in 2D space. Each dot represents one cell image and is colored by its matching cell line name. There were clear gaps between different cell lines, which validates that CLCNet model can distinguish the 30 cell lines well. Example images of the 30 cell lines are shown in Fig. S6.

**Figure 5:**
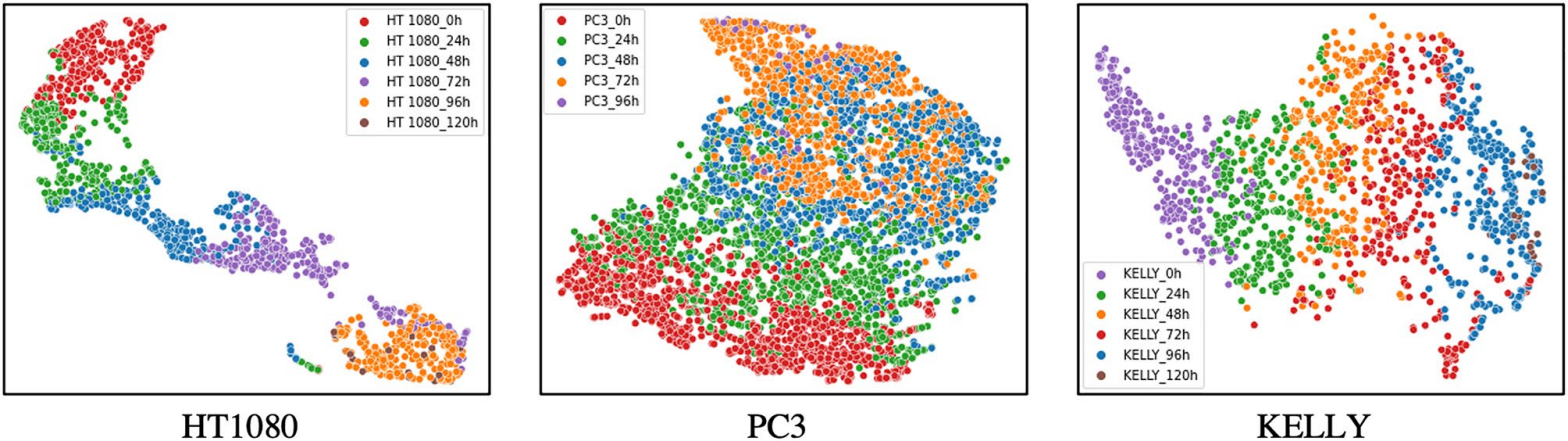
t-SNE plots of 3 example cell lines (e.g. HT1080, PC3, KELLY). These plots enlarge the three cell lines’ distribution of Fig. 4. Each dot is colored by the range of the incubation duration (e.g. 0–24 h, 24–48 h). An interesting phenomenon was found that samples with similar times locate in adjacent areas. For example, the samples of HT1080 were clustered well and samples within 0–24 h were close to the samples within 24–48 h.

To further view how the deep network distinguished the 30 cell lines, we used t-SNE embedding to reduce the dimension of the convolutional features of CLCNet (e.g. 2048 dimensions) and visualized the processed features in 2d space. The t-SNE plot of the 30 cell lines is shown in Fig. 4. Each dot represents one cell image and is colored by its cell line label. There were clear gaps between different cell lines which validates that CLCNet can find the decision boundary and distinguishes the 30 cell lines well. When we associated the cell lines’ distribution with their incubation times, we found an interesting phenomenon that samples with similar times are mapped to adjacent areas. For example, in Fig. 5, the samples of HT1080 were clustered well. Samples within 0–24 h were close to the samples within 24–48 h. The complete results of the 30 cell lines with their incubation times are shown in Fig. S3.

### Identify new cell lines

**Figure 6:**
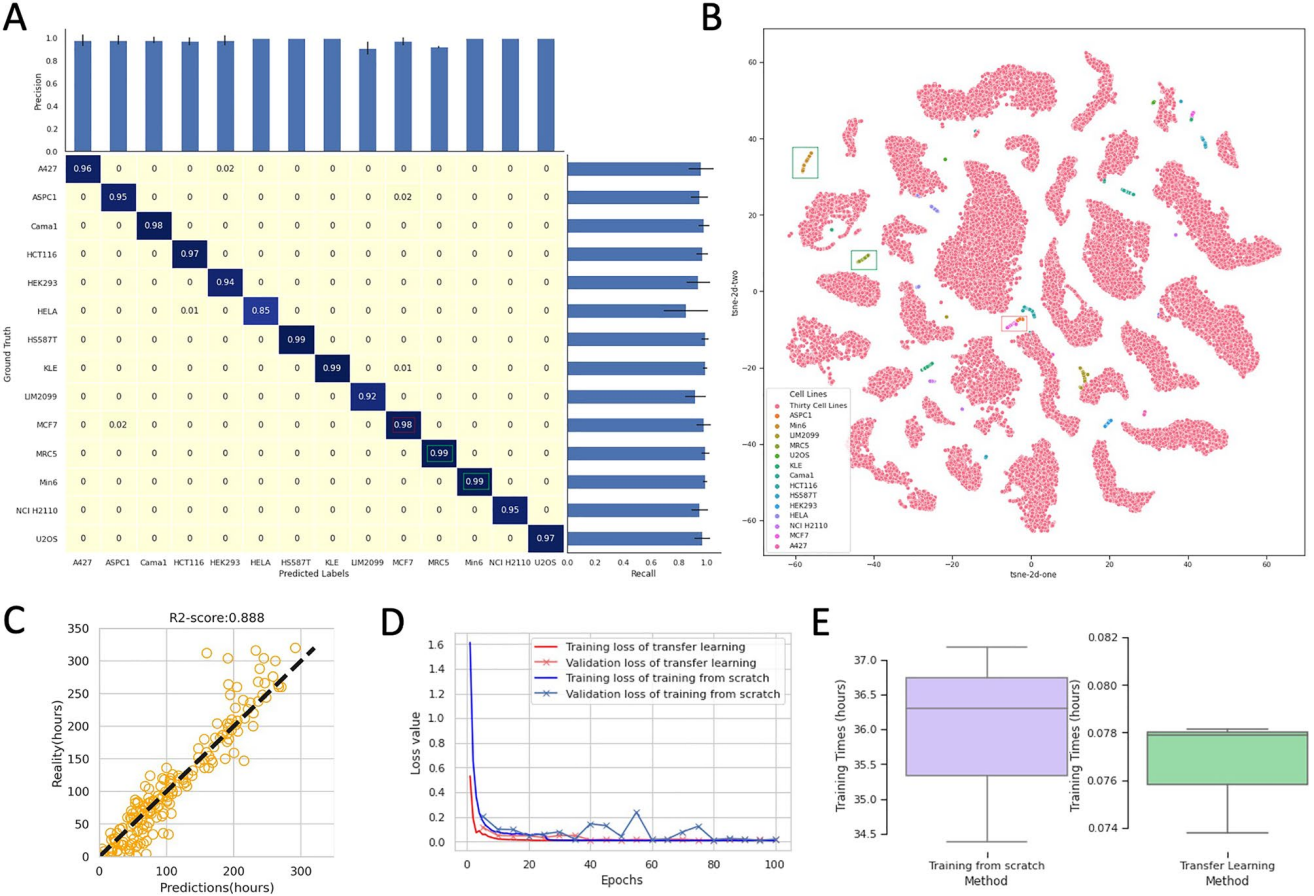
Performance of the transfer learning technique for identifying 14 new cell lines. (**A)** Confusion matrix of the 14 cell line classification. (**B)** t-SNE plot of 44 cell lines. The pink dots are the data of 30 cell lines and dots of other colors are the data of 14 new cell lines. It can be seen that the samples of the 14 new cell lines were mapped to the margin space between the 30 cell lines. Example images of the 14 cell lines are shown in Fig. S7. (**C)** Regression results for the test fold-1 of the cross-validation. (**D)** Comparison of the convergence speed between full model training from scratch and final layer fine-tuning using the transfer learning. “Training from scratch” means that all layers of Xception are re-trained using the dataset. (**E)** Comparison of the training times.

The confusion matrix of the transfer learning technique for identifying 14 new cell lines is shown in Fig. 6A. It is clear that integrating the transfer learning with CLCNet can identify 14 new cell lines well with 96.5% accuracy. We showed the t-SNE plot of the 44 cell lines in Fig. 6B. The pink dots show the data for the 30 cell line dataset and the additional colors show the 14 new cell line samples. The samples of the 14 new cell lines were mapped to the margin space between the 30 cell lines. By comparing Fig. 6A with Fig. 6B, some misclassified cases can be explained. For example, in Fig. 6A, 98% of MCF7 images were classified accurately but 2% of data were misclassified as ASPC1. In Fig. 6B, some dots of MCF7 (i.e. red box) gathered with the ASPC1’s dots which represents that the feature vectors of MCF7 images are similar to that of ASPC1. In contrast, the well-classified cases (i.e. green box) MRC5 and Min6 clustered well in the t-SNE plot and obtained the accuracy of 99%. We showed the regression results for the test fold-1 of the 14 cell lines in Fig. 6C and the whole cross-validation results were presented in Fig. S4.

We demonstrated the advantages of the transfer learning method compared with the way of training model from scratch in Fig. 6D,E. The transfer learning method requires less computational resource and considerably less training time compared with training the whole model from scratch. The transfer learning method only spent four training epochs to loss convergence and its average training time is 0.078 h. The overall performance of the transfer learning technique for identifying the 44 cell lines was shown in Table 2.

**Table 2:** Classification and regression results of the transfer learning technique for identifying the 44 cell lines.

Metrics	14 cell lines	30 cell lines	Overall
Accuracy	0.965 $\pm$ 0.018	0.998 $\pm$ 0.001	0.997 $\pm$ 0.001
Precision	0.977 $\pm$ 0.012	0.997 $\pm$ 0.001	0.991 $\pm$ 0.004
Recall	0.958 $\pm$ 0.024	0.996 $\pm$ 0.001	0.984 $\pm$ 0.008
F1-score	0.968 $\pm$ 0.017	0.997 $\pm$ 0.001	0.987 $\pm$ 0.006
MSE	526.230 $\pm 62$.090	232.690 $\pm 0.000$	263.360 $\pm 3.287$
R2-score	0.853 $\pm$ 0.017	0.939 $\pm$ 0.000	0.932 $\pm$ 0.001

The columns of the “14 cell lines” represented the data of 14 cell lines is split from the test dataset during evaluation. The columns of the “30 cell lines” represented the data of 30 cell lines is split from the test dataset during evaluation. The “overall” column showed the results for the whole test set (44 cell lines).

## Discussion

In summary, in the classification task of identifying 30 cell lines, our proposed computer-aided system CLCNet achieved the overall performance of 99.7% precision and the recall of 99.7%. We compared the Xception backbone with three SOTA architectures, e.g. ResNet50, VGG19, MobileNet. The classification performance of the three deep networks has been validated in the previous studies on the analysis of cellular images^10,15,16^. In our experiments, the Xception model outperformed the other architectures, which can be attributed to the DSC blocks which decouple spatial correlations from cross-channel correlations and allows more efficient training. It should be noted that MobileNet also includes several DSC layers in its structure^26^, however MobileNet is lightweight and targets the deployment on a mobile platform ahead of outright performance. Hence, we picked up the Xception as the backbone of CLCNet for classification. We used the t-SNE plots (Fig. 4) to visualize the high-dimensional features of CLCNet. It is clear that samples (dots) that belong to the same cell lines are clustered together and there are clear gaps between the 30 cell lines. These results proved that CLCNet is powerful for identifying the 30 cell lines.

The regression performance of CLRNet was satisfactory with an R2-score = 0.931. We also conducted an ablation experiment using the cell images to train Xception purely for regression. Here, the Xception architecture is almost the same as Fig. S1 except for the final FC layer. The FC only includes one neuron and the used loss function is MSE. The regression results of the ablation model are shown in Fig. S5 and the performance is really poor. Because in our dataset, some cell images from different cell lines have the same time labels which affects the training of the network. For example, we collected two cell images from A431 and A549 cell lines separately but these two images have the same incubation times (e.g. Fig. 1). There are many differences between the two cell images, e.g. the single cell morphology, cell counts, and confluency. The two images have completely different appearances but take the same time labels, which influences the feature learning of the network. From Fig. 5, we observed that the convolutional features of CLCNet include information about the incubation times. Hence, we took the features of CLCNet as the input of CLRNet to predict the incubation times for cell images. The above results validated our strategy is effective for the regression task.

Aiming to handle data from new cell lines, we integrated the transfer learning technique with our proposed framework. We established a small-scale dataset that included 14 cell lines to evaluate the method’s performance. From the pre-trained model side, the 14 new cell lines were unseen so the model cannot output the matching labels for these images. Considering the pre-trained CLCNet has learned good representations from the data of 30 cell lines, we fixed the parameters of all layers except for the final FC layer. The FC were re-trained by using the combined dataset (i.e. training set from 30 cell lines + training set from 14 cell lines). The classification accuracy for the 14 cell lines reached 96.5% and the performance of identifying the 30 cell lines was not influenced (99.8% accuracy) by re-training. Although the transfer learning technique needs to re-train the part of CLCNet, its training speed was 480 times faster than training the model from scratch. These results suggested that the transfer learning method is possible to solve the issue.

In this paper we have established a proof of concept for how an image-based AI method can be used for cell line authentication, including the use of transfer learning to extend classification to cell lines for which we have only a small number of example images. In doing so we have identified a number of areas for future research. Firstly, the ability to identify and quantify contamination of cell lines with small amounts of another cell line would be a useful functionality. Secondly, having to retrain the model when adding new cell lines might be a drawback as greater numbers of cell lines are built up. Some new techniques like conformal prediction^27^ or open set recognition^28^ can automatically decide data is seen or unseen without extra model training. These methods can potentially be integrated with the transfer learning method to solve this issue. Besides, flagging or analyzing the growth processes of stem cells will be an application direction of our proposed regression model. There is also the question of how much training data is necessary: How many cell lines are required for the base training (is 30 cell lines enough)? How many cell lines can this be expanded to via transfer learning (200 cell lines or more) whilst maintaining a satisfactory level of performance? Finally, we have demonstrated a proof of concept for rapid cell line authentication. When this system is deployed into the laboratory, the development of guidelines for interpreting the confidence value outputted by CLCNet, and the incubation time predicted by CLRNet, how to assess performance and how to integrate with current laboratory practice are required.

In this study, we have attempted to automatically authenticate cell lines by using deep neural networks on brightfield images. We proposed a novel multi-task cell image recognition framework to authenticate cell lines and predict the duration of incubation. We established a large dataset consisting of 47,671 brightfield images of 30 cell lines. The classification and regression performance on the dataset were excellent. We also integrated transfer learning with our proposed system to identify new cell lines and obtained 96.7% accuracy. These results demonstrated that our proposed framework can effectively authenticate the identities of cell lines on brightfield images.

## Supplementary Information

Supplementary Figures.

Supplementary Table S1.

## Data Availability

The datasets generated and/or analyzed during the current study are not publicly available due AstraZeneca Licenses but are available from the corresponding author on reasonable request. Source code from this project and our model can be accessed from GitHub: https://github.com/BIPL-UoL/An-Automated-Cell-Line-Authentication-Method-for-AstraZeneca-Global-Cell-Bank.
