# Supplementary material for: An automated cell line authentication method for AstraZeneca global cell bank using deep neural networks on brightfield images
Source: Sci Rep. 2022 May 12;12:7894. doi: 10.1038/s41598-022-12099-3 (PMC9098893; doi:10.1038/s41598-022-12099-3)
Supplement: Supplementary file 1 — Supplementary Figures. [file 41598_2022_12099_MOESM1_ESM.pdf]

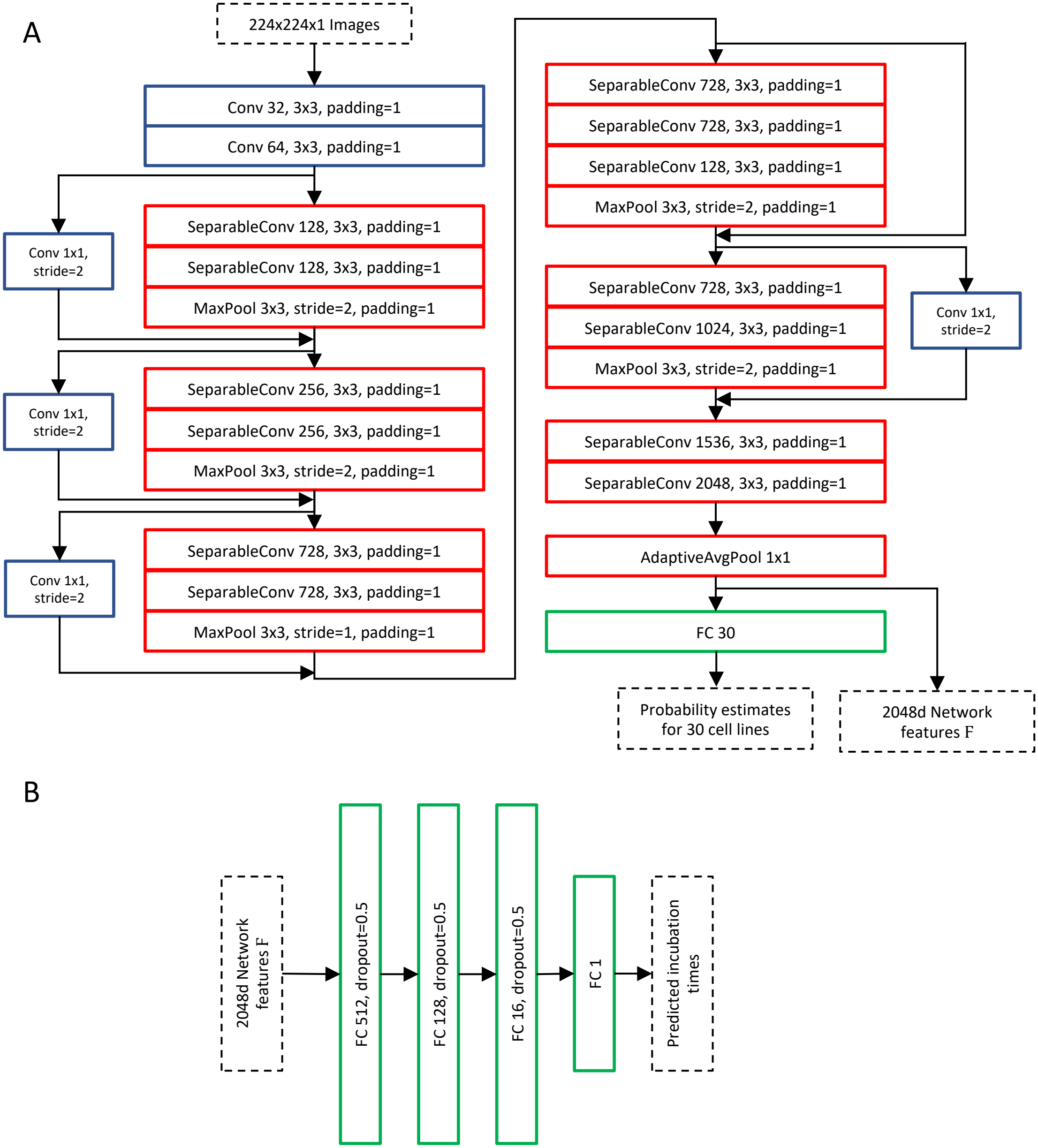

Fig. S1 Architectures of CLCNet and CLRNet. A. CLCNet structure. B. CLRNet structure. Conv is the convolution layer, BatchNorm is the batch normalization, ReLU is the activation function, SeparableConv is the depthwise separable convolution layer, MaxPool is the Maxpooling layer, and FC is the fully connected layer.

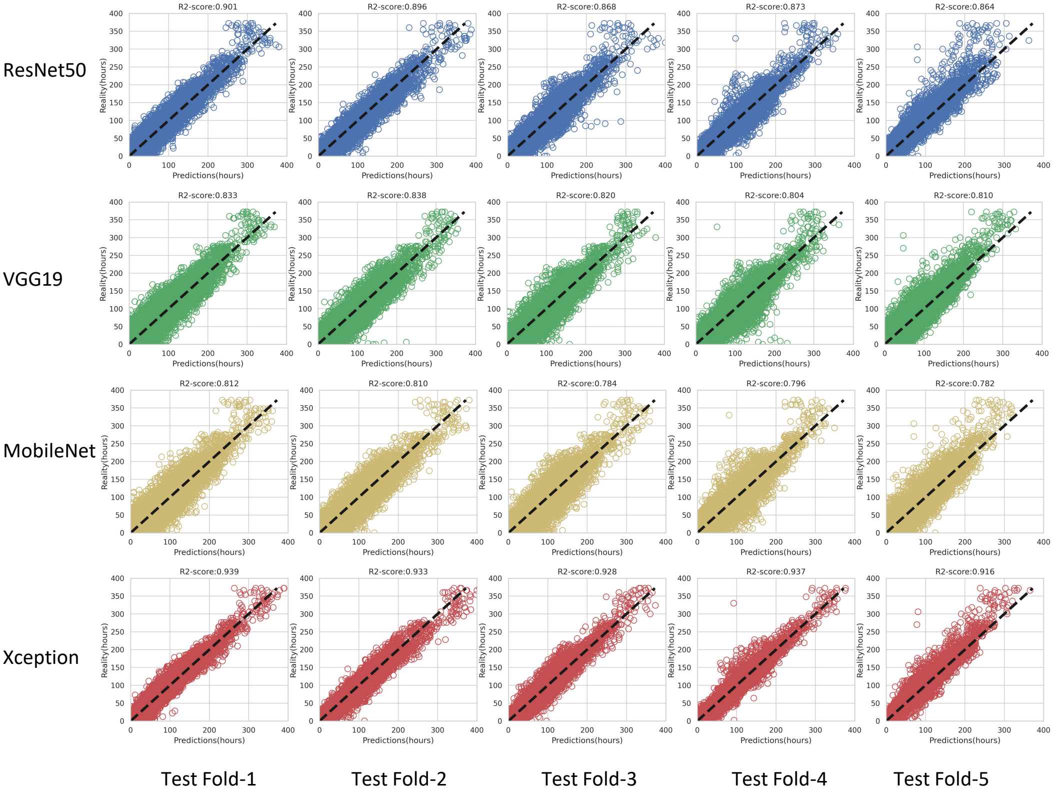

Fig. S2 Regression Results of four backbones(e.g. ResNet50, VGG19, MobileNet, Xception) on 5-fold Cross-Validation. Scatter plots of the predicted incubated durations vs. the real incubated durations.

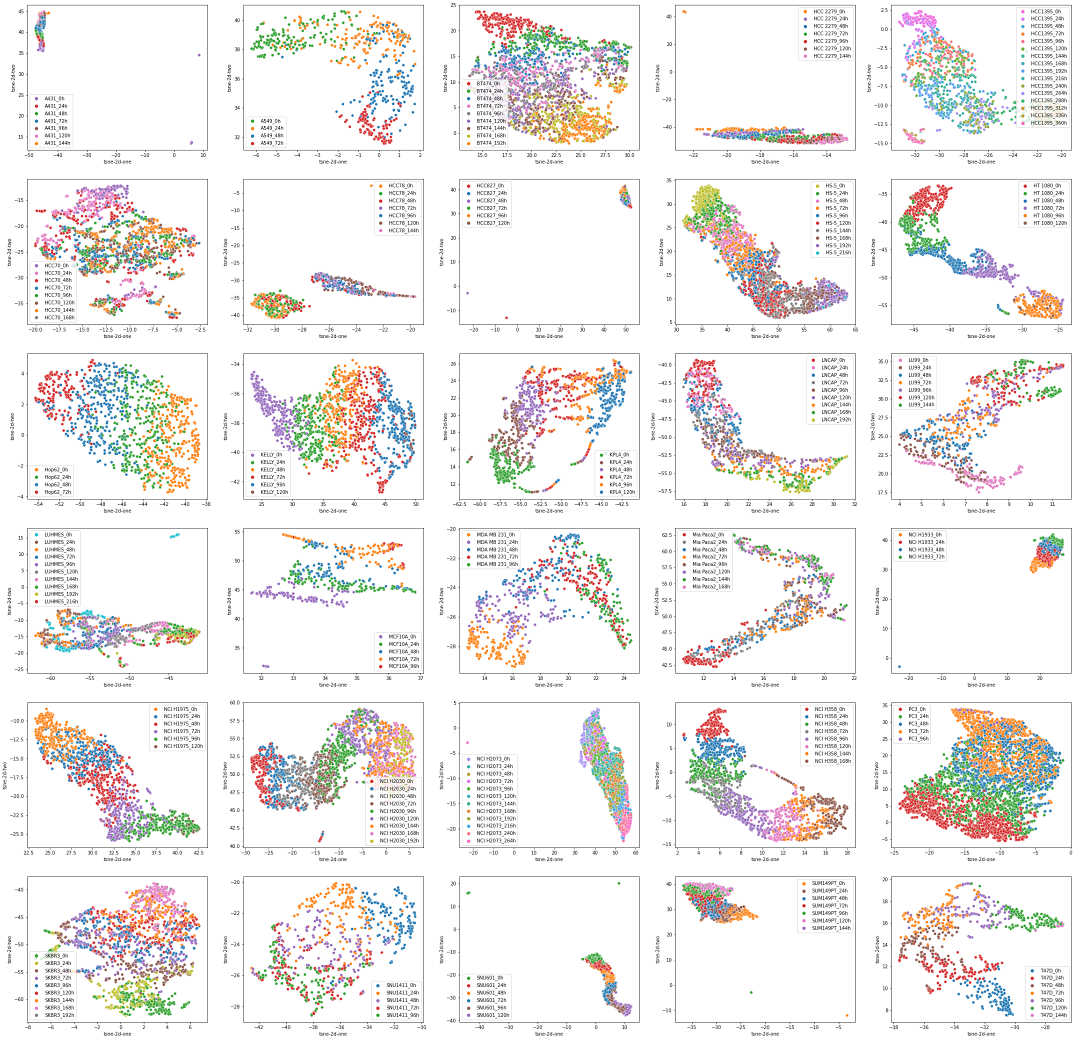

Fig. S3 TSNE plots of 30 cell lines. Each dot is colored by the range of the incubation duration (e.g. 0-24 hours, 24-48 hours).

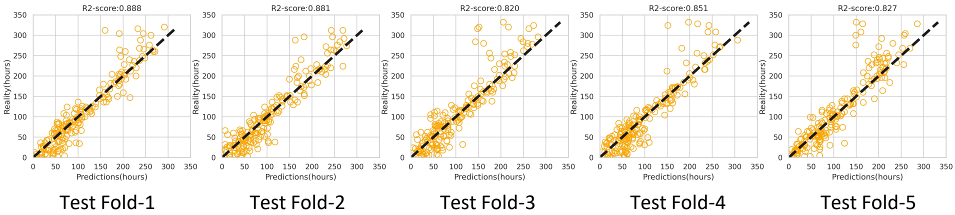

Fig. S4 Regression Results of transfer learning method for 14 new cell lines on 5-fold Cross-Validation. Scatter plots of the predicted incubated durations vs. the ground truth incubated durations.

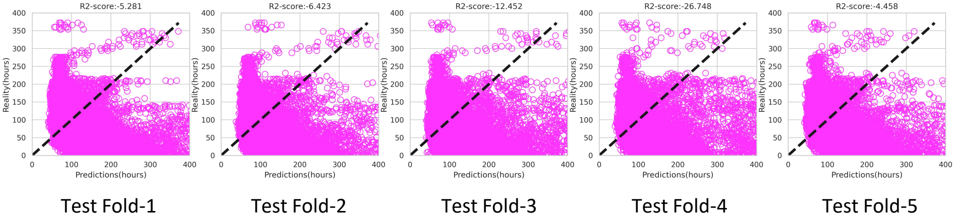

Fig. S5 Failure cases of training Xception purely with raw cell images for regression.

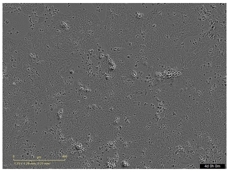
A431

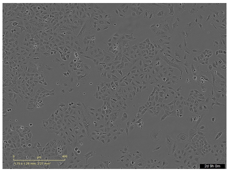
A549

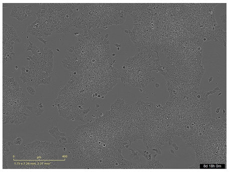
BT474

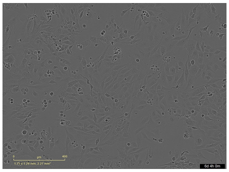
HCC2279

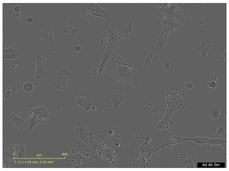
HCC70

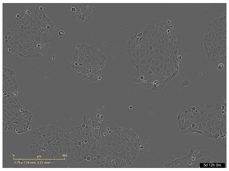
HCC78

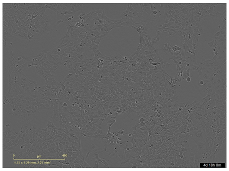
HCC827

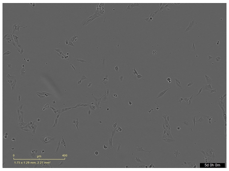
HCC1395

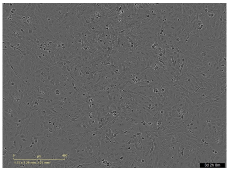
Hop62

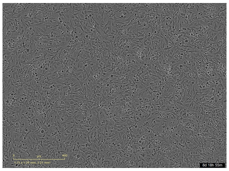
HS-5

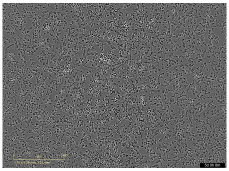
HT1080

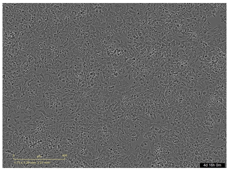
KELLY

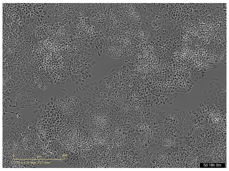
KPL4

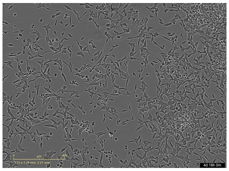
LNCAP

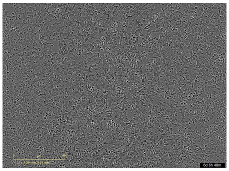
LU99

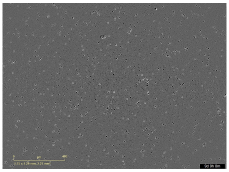
LUHMES

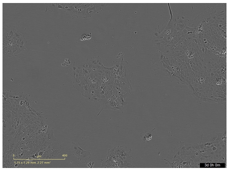
MCF10A

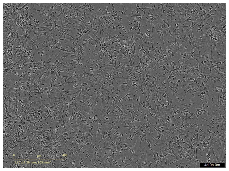
MDA MB 231

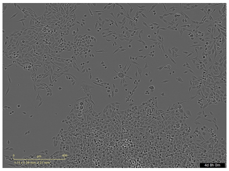
Mia Paca2

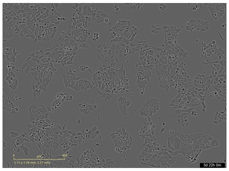
NCI H358

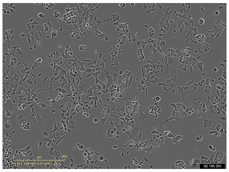
NCI H1933

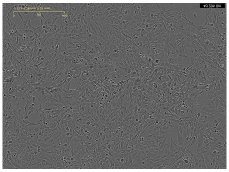
NCI H1975

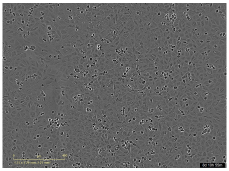
NCI H2030

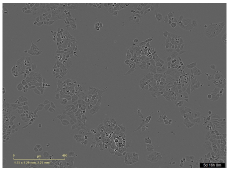
NCI H2073

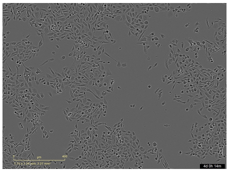
PC3

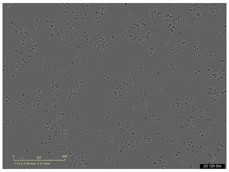
SKBR3

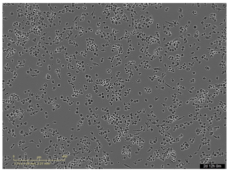
SNU601

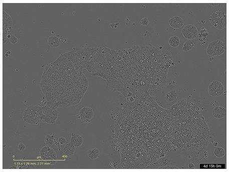
SNU1411

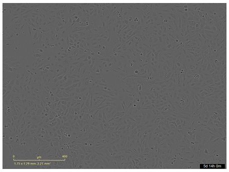
SUM149PT

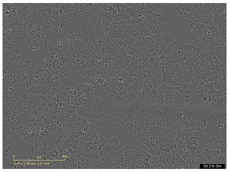
T47D

Fig. S6 Example image of 30 cell lines

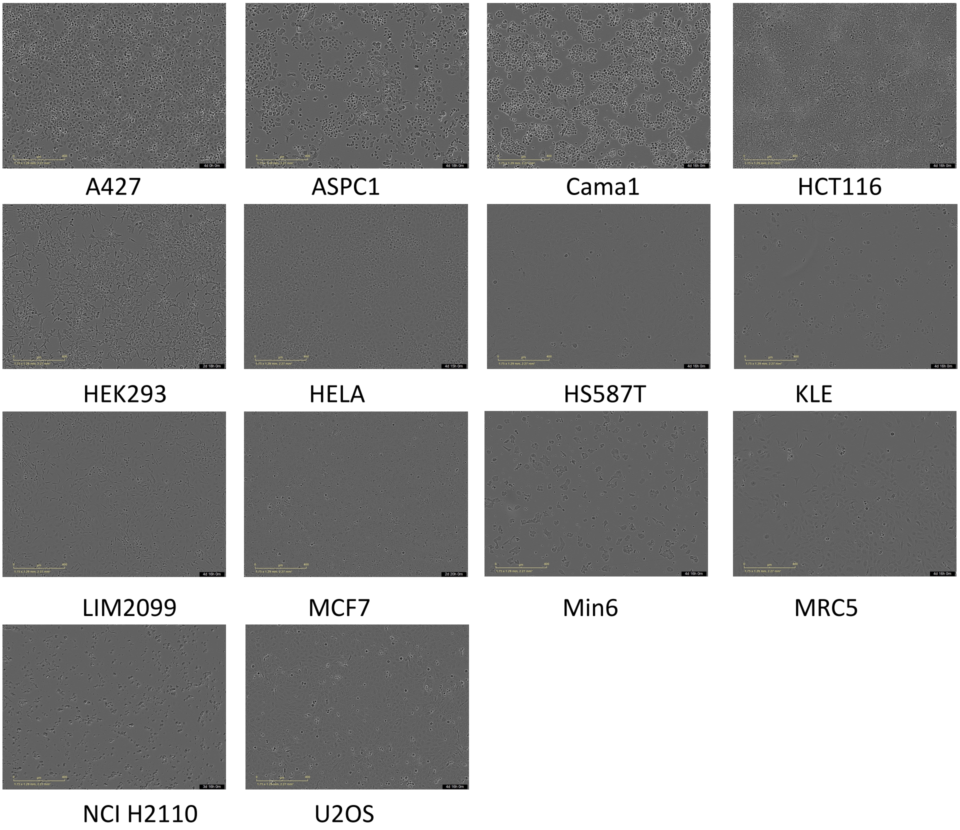

Fig. S7 Example image of 14 new cell lines
