# Supplementary material for: An automated cell line authentication method for AstraZeneca global cell bank using deep neural networks on brightfield images
Source: Sci Rep. 2022 May 12;12:7894. doi: 10.1038/s41598-022-12099-3 (PMC9098893; doi:10.1038/s41598-022-12099-3)
Supplement: Supplementary file 2 — Supplementary Table S1. [file 41598_2022_12099_MOESM2_ESM.pdf]

Table. S1 List of 30 cell lines. All base medium was purchased from Sigma Aldrich, and supplemented with 10% FBS (Sigma Aldrich) and 1x GlutaMAX (Gibco) unless otherwise stated.

Cell Line	Source	Base Medium	Image Number
A431	ATCC	Dulbecco's Modified Eagle Medium	370
A549	ATCC	RPMI 1640	580
BT474	ATCC		2700
HCC 2279	KCLB	Dulbecco's Modified Eagle Medium	800
HCC1395	ATCC	RPMI 1640	1258
HCC70	ATCC	RPMI 1640	2225
HCC78	DSMZ	RPMI 1640	720
HCC827	ATCC	RPMI 1640	420
HS-5	ATCC	Dulbecco's Modified Eagle Medium	2925
HT 1080	ATCC	Eagle's Minimum Essential Medium, 1% NEAA	1525
Hop62	NCI	RPMI 1640	1075
KELLY	DSMZ	RPMI 1640	1525
KPL4	Kawasaki Medical Centre	RPMI 1640	1440
LNCAP	ATCC	RPMI 1640	1134
LU99	JCRB	RPMI 1640	820
LUHMES	ATCC	1:1 Mix of DMEM and Ham's F-12, N2 Supplement 15, bFGF 40 ng/mL	1560
MCF10A	ATCC	DMEM / F-12 (1:1 Mix of DMEM and Ham's F-12), Horse Serum 5%, Cholera Toxin 0.1 ug/ml, EGF 20 ng/mL, Glutamax 15, Hydrocortisone	520

		0.5 ug/mL, Insulin 10 ug/mL	
MDA MB 231	ATCC	RPMI 1640	780
Mia Paca2	ECACC	Dulbecco's Modified Eagle Medium	920
NCI H1993	ATCC	RPMI 1640	929
NCI H1975	ATCC	RPMI 1640	1525
NCI H2030	ATCC	RPMI 1640	2800
NCI H2073	ATCC	RPMI 1640	2549
NCI H358	ATCC	RPMI 1640	1920
PC3	ATCC	Ham's F12, Kaighn's Modification	4836
SKBR3	ATCC		1940
SNU1411	KCLB	RPMI 1640	760
SNU601	KCLB	RPMI 1640	1400
SUM149PT	Asterand Bioscience/BioIVT	Ham's F12, FBS 5%, Glutamax 1%, Hydrocortisone 0.5 ug/mL,Insulin 10 ug/mL	1410
T47D	ATCC	RPMI 1640	830

Table. S2 List of 14 cell lines.

Cell Line Name	Source	Base Medium	Image Number
A427	ATCC	EMEM	25
ASPC1	ATCC	RPMI	39
Cama1	ATCC	EMEM	96
HCT116	ATCC	McCoys 5A	69
HEK293	ATCC	EMEM	34
HELA	ATCC?	EMEM	55
HS578T	ECACC	DMEM, 0.01mg/ml Insulin	84
KLE	ATCC	DMEM:Hams F12	84
LIM2099	ECACC	RPMI, 10uM 1-Thioglycerol, 1ug/ml Hydrocortisone, 0.5ug/ml Insulin	84
MCF7	ATCC	EMEM, 1% NEAA	69
MRC5	ECACC	EMEM, 1% NEAA	140
Min6		DMEM, 50uM 2-Mercaptoethanol	45
NCI H2110	ATCC	RPMI	36
U2OS	ATCC	McCoys 5A	36
